# Association of *ESR1* (rs2234693 and rs9340799), *CETP* (rs708272), *MTHFR* (rs1801133 and rs2274976) and *MS* (rs185087) polymorphisms with Coronary Artery Disease (CAD)

**DOI:** 10.1186/s12872-020-01618-7

**Published:** 2020-07-18

**Authors:** Jyotdeep Kour Raina, Minakashee Sharma, Rakesh Kumar Panjaliya, Vikas Dogra, Ashok Bakaya, Parvinder Kumar

**Affiliations:** 1 Institute of Human Genetics, University of Jammu, Jammu, JandK 180006 India; 2 Human Genetics Lab, Department of Zoology, University of Jammu, Jammu, JandK India; 3 Department of Zoology, Government Degree College, Samba, JandK India; 4 Department of Cardiology, Acharaya Shri Chander College of Medical Sciences (ASCOMS) and Hospital, Sidhra, Jammu, JandK India

**Keywords:** CAD, *CETP*, *ESR1*, *MTHFR*, *MS*, Polymorphism

## Abstract

**Background:**

Coronary artery disease (CAD) is a complex disease resulting from the cumulative and interactive effects of large number of genes along with environmental exposure. Therefore, the present study was envisaged as an effort to study the association of candidate genes *ESR1* (rs2234693 and rs9340799), *CETP* (rs708272), *MTHFR* (rs1801133 and rs2274976) and *MS* (rs185087) polymorphisms with the risk of CAD, targeting the populations of Jammu (JandK).

**Method:**

A total of 400 confirmed CAD patients and 400 healthy controls were enrolled for the present study. Genotyping was done by polymerase chain reaction- restriction fragment length polymorphism (PCR-RFLP).

**Results:**

*ESR1* gene (rs9340799) polymorphism was found to be associated with CAD in all the genetic models. The haplotype analysis of *ESR1* (rs2234693 and rs9340799) gene revealed that C-G haplotype was conferring approximately 5-fold risk and T-A haplotype was adding 1.4-fold risk towards the disease. ‘T’ allele of *MTHFR* rs1801133 SNP was observed to be responsible for development of CAD in our study population (*p* < 0.0001). In case of *MTHFR* (rs1801133 and rs2274976) gene, the haplotype T-G was observed to confer 4.7-fold risk towards CAD whereas haplotype C-G provided nearly a 1.7 fold protection towards development of CAD. For *MS* gene, rs185087 was also found to be associated with CAD in a co-dominant (*p* = 0.003 and *p* = 0.03), dominant (*p* = 0.001) and allelic models (p = 0.001). The gene-gene interaction revealed strong epistasis between single nucleotide polymorphisms (SNPs), *ESR1* rs9340799 and *MTHFR* rs2274976. Furthermore, the dendrogram for gene-environment dataset indicated moderately synergistic interaction between *CETP* rs708272 and physical inactivity.

**Conclusion:**

In the study under reference, a significant association of *ESR1*-*XbaI* (rs9340799), *MTHFR* C677T (rs1801133) and *MS* A2756G (rs185087) gene polymorphisms with the susceptibility of CAD in the population of Jammu region (JandK) has been observed.

## Background

Cardiovascular diseases (CVDs) have become a leading global cause of death, accounting for more than 17.6 million deaths in 2016 and the number is expected to grow more than 23.6 million by 2030 [1]. The plausible justification for increase in CVD cases in India is that presently the nation is facing rapid urbanisation resulting in changing lifestyle. In addition, health damaging activities such as tobacco use, consumption of high caloric food and stress are also playing a potential role in increasing CVD cases.

Coronary artery disease (CAD) is a major CVD phenotype being noticed in Indians. It is estimated that Asian Indians constitute a fifth of the global population with CAD [2]. CAD is a complex disease resulting from the cumulative and interactive effects of large number of genes along with environmental exposure.

The female reproductive hormone estrogen is also well known to affect cardiovascular functions, such as maintenance of lipid profile, anti-atherosclerotic and anti-inflammatory action, increasing vasodilation, enhancing HDL levels, attenuation of cardiac cell apoptosis [3, 4] and modulation of insulin secretion [5]. The hormone binds with a ligand activated transcription factor, estrogen receptor alpha (ERα) encoded by *ESR1* gene and influences several physiological and cellular processes. The biological role of *ESR1* gene can be mutated due to the presence of polymorphic variations in intron 1 (rs2234693 and rs9340799) and is associated with dyslipidemia, MI and CAD [6, 7].

Cholesteryl ester transfer protein (CETP) encoded by *CETP* gene on 16q21 mediates the exchange of lipids between anti-atherogenic high-density lipoprotein (HDL) and atherogenic apolipoprotein (apo) B containing lipoproteins and, therefore, plays a major role in lipid metabolism. *TaqIB* polymorphism (rs708272) at the *CETP* locus is associated with changes in lipoprotein size, CETP activity and HDL-C levels [8].

Homocysteine (Hcy), a toxic sulphur-containing amino acid formed during methionine metabolism is known to have atherogenic nature. Two genes namely Methylenetetrahydrofolate reductase (*MTHFR*) on chromosome 1p36.3 and Methionine synthase (*MS/MTR*) on 1q43 are considered to be critical in lowering circulatory homocysteine levels [9]. *MTHFR* catalyses the conversion of 5, 10- methylenetetrahydrofolate (5,10-MTHF) into 5-methyltetrahydrofolate (5-MTHF). The methyl group of 5-MTHF is given to Hcy to form methionine by *MS* gene via remethylation reaction [9].

Given the close association between atherogenic factors (toxic homocysteine and altered lipid levels) and CAD susceptibility, with various polymorphisms in the genes mentioned, the study of these SNPs viz. *ESR1* (rs2234693 and rs9340799), *CETP* (rs708272), *MTHFR* (rs1801133 and rs2274976) and *MS* (rs185087) in North Indian population of Jammu region of the Jammu and Kashmir UT may be of interest. However, there is no comprehensive data available that allows comparison of genetic factors in context to CVD susceptibility from the population of Jammu region.

## Methods

### Subjects

The current study has been carried out on 400 confirmed cases of CAD and 400 unrelated healthy controls belonging to different areas of Jammu region of Union Territory of Jammu and Kashmir (the erstwhile Jammu and Kashmir State). Clinically confirmed CAD patients (confirmed by coronary angiography: > 50% stenosis in at least one of the arteries) along with episode of MI and history of hypertension were enrolled from Out Patient Department of Cardiology, Acharaya Shri Chander College of Medical Sciences (ASCOMS) and Hospital, Sidhra, Jammu and private clinics (from 2015 to 2017). The diagnosis of MI was based on typical electrocardiographic changes and on raised levels both in the serum activities of enzymes such as creatine kinase, aspartate aminotransferase, and lactate dehydrogenase and in the serum concentration of troponin T. The controls were recruited from hospital staff; staff of University of Jammu and individuals with minor unrelated ailments attending hospital. The control subjects had no history of MI or CAD, stroke, other atherosclerotic diseases or other embolic, thrombotic, or hemorrhagic disorders or metabolic disease. Individuals with primary hypertension and dyslipidemia were also excluded. The present study design was duly approved by Animal and Human Experimentation Ethical Committee (AHEEC), University of Jammu. Data and blood collection was done after having an informed written consent from each study participant.

### Samples and data collection

A detailed, pre-designed health questionnaire, including parameters such as age, gender, dwelling, habit of smoking, parameters of physical inactivity along with anthropometric and physiometric variables was duly filled based on inputs from each individual. Body mass index (BMI) was calculated as ratio of weight and height (weight in kg and height in m^2^) and the values were defined according to the recommendations proposed by WHO for Asians [10]. Waist Hip ratio (WHR) was obtained as waist circumference divided by hip circumference and was defined as ≥0.89 for men and ≥ 0.81 for women. Pulse rate (PR) was counted by feeling radial artery at the wrist over one minute. Pulse pressure (PP) was calculated by applying formula: PP = Systolic blood pressure (SBP) - Diastolic blood pressure (DBP) [9]. Three milliliters of peripheral blood was collected in EDTA vacutainers from each fasting study individual. Lipid profiling was done on automated biochemical analyser (Roche, Cobas CIII). The diagnostic criteria for dyslipidemia included abnormal lipid levels with serum triglyceride level ≥ 150 mg/dl, high total cholesterol level ≥ 200 mg/dl, high Low density lipoprotein cholesterol (LDL-C) level ≥ 130 mg/dl or low High density lipoprotein- cholesterol (HDL-C) level < 40 mg/dl and patients on lipid lowering drugs at the time of the study [11]. According to Joint National Committee-7 (JNC 7) guidelines patient on antihypertensive medications or having a systolic blood pressure (SBP) of 140 mmHg or greater and a diastolic blood pressure (DBP) of 90 mmHg or greater were considered as having hypertension [12].

### Genotyping of the selected polymorphisms

Genomic DNA was extracted from whole blood samples using Phenol-chloroform isoamyl alcohol method with slight modifications [13]. Isolated genomic DNA was stored at − 20 °C until the genotyping was done. The PCR amplification profiles for selected polymorphisms along with their restriction enzymes are given in Table 1. For validation of genotype results 50 samples selected randomly each from cases and controls were duplicated for PCR-RFLP analysis and were found to be free of false positives.

**Table 1:** Candidate gene polymorphisms, their primer sequence and restriction digestion products

SNP No.	Gene polymorphism	Primer sequence	Amplicon (bp)	Restriction enzymes	Genotypes
SNP 1	*ESR1* IVS1–397 T/C (rs2234693)	5′-CTG CCA CCC TAT CTG TAT CTT TTC CTA TTC TCC- 3′ (F) 5′-TCT TTC TCT GCC ACC CTG GCG TCG ATT ATC TGA- 3′ (R)	1372	*PvuII*	TT = 982 & 390 bp TC = 982, 390 & 1372 bp CC = 1372 bp
SNP 2	*ESR1*- IVS1–351 A/G (rs9340799)	5′-CTG CCA CCC TAT CTG TAT CTT TTC CTA TTC TCC- 3′ (F) 5′-TCT TTC TCT GCC ACC CTG GCG TCG ATT ATC TGA- 3′ (R)	1372	*XbaI*	AA = 936 & 436 bp AG = 936, 436 & 1372 bp GG = 1372 bp
SNP 3	*CETP*-TaqIB (C277T) (rs708272)	5′-CAC TAG CCC AGA GAG AGG AGT GCC-3′ (F) 5′-CTG AGC CCA GCC GCA CAC TAA C-3′ (R)	535	*TaqI*	B2B2 = 535 bp B1B2 = 535, 361 & 174 bp B1B1 = 361 & 174 bp
SNP 4	*MTHFR* C677T (rs1801133)	5′-TGA AGG AGA AGG TGT CTG CGG GA-3′ (F) 5′-AGG ACG GTG CGG TGA GAG TG-3′ (R)	198	*HinfI*	CC = 198 bp CT = 198, 175 & 23 bp TT = 175 & 23 bp
SNP 5	*MTHFR* G1793A (rs2274976)	5′-CTC TGT GTG TGT GTG CAT GTG TGC G-3′ (F) 5′-GGG ACA GGA GTG GCT CCA ACG CAG G-3′ (R)	310	*BsrbI*	GG = 233 & 77 bp GA = 310, 233 & 77 bp AA = 310 bp
SNP 6	*MS* A2756G (rs185087)	5′- TGT TCC AGA CAG TTA GAT GAA AAT C-3′ (F) 5′- GAT CCA AAG CCT TTT ACA CTC CTC-3′ (R)	211	*HaeIII*	AA = 211 bp AG = 211, 131 & 80 bp GG = 131 & 80 bp

### Statistical analysis

For non- genetic variables, Mean and Standard deviation were calculated and Student’s t-test was performed to calculate the difference between the patients and the controls at 0.05 level of statistical significance. Genotypic as well as allelic frequencies were calculated by gene counting method. Hardy-Weinberg equilibrium (HWE) analysis and the differences in genotypic frequencies between two study groups were examined by using Pearson’s goodness of fit Chi-square test. To assess the association of CAD risk, odds ratios (OR) with 95% CI were calculated at 0.05 level of statistical significance under different genetic models by using Statistical Package for Social Sciences (SPSS-version 20) software. The power of the study was calculated using the CaTS power calculator for one-stage genetic association studies [14] (http://www.sph.umich.edu/csg/abecasis/CaTS/index. html). The power for the SNPs viz. *ESR1* (rs2234693 and rs9340799), *CETP* (rs708272), *MTHFR* (rs1801133 and rs2274976) and *MS* (rs185087) was found to be 98, 99%, 99, 66, 86 and 96% respectively. The pair wise linkage disequilibrium (LD) and its respective measures (D’, LOD and r^2^) for the *ESR1* and *MTHFR* SNP’s among cases and controls were calculated by using Haploview 4.2 version software based on expectation-maximization (EM) algorithm (http://haploview.software.informer.com/4.2/). The colour code on plot follows the standard colour scheme for Haploview viz. for white D’ < 1, LOD < 2; for shades of pink/red D’ < 1, LOD ≥ 2; for blue D’ = 1, LOD < 2 and for bright red D’ = 1, LOD ≥ 2. Interpretation of the interaction between selected polymorphisms, environmental exposure and with the disease was studied through interaction dendrograms generated by Multifactor Dimensionality Reduction (MDR) (version 3.0.2) as prescribed by Ritchie et al.*,* [15]. MDR program is designed to test for interactive genetic and non-genetic effects on a trait even if the independent effects are non-significant. Among the sets of Multifactor models, the combinations of genotypes that showed maximum testing balance accuracy (TBA) and the highest cross-validation consistency (CVC) were chosen. CVC is defined as the number of times a particular interaction model is selected across 10 cross-validation datasets, with the corresponding *p*-value. Statistical significance was evaluated using a 1000 permutation test to compare observed TBA.

## Results

### Non-genetic factors

Associations of different non-genetic parameters with CAD were studied and have been presented in Table 2. Maximum disease load was observed in patients from urban dwellings. Frequency of CAD cases reported from urban dwellers counterparts was 53.75% and that from rural counterparts of Jammu was 46.25%. BMI and WHR were significantly higher in patients than in controls (*p* < 0.0001 and *p* = 0.008, respectively). The mean SBP and DBP indicated significant (p < 0.0001) differences in trait variance among the two study groups. A significant difference was observed between PP (53.73 ± 16.77 vs 41.58 ± 8.12, *p* < 0.0001) and PR (82.11 ± 12.23 vs 74.41 ± 4.91, p < 0.0001) values in patients and controls as also in metabolic variables like TC, TG, LDL and HDL levels among the study participants. The prevalence of smoking was higher in patients than in controls. The parameter of physical inactivity has also been found to be another prevalent risk factor associated with CAD in our study [OR = 2.40, 95% CI (1.80–3.18), p < 0.0001].

**Table 2:** Association of Non-genetic variables in the study subjects

Parameters	Patients (***N*** = 400)	Controls (***N*** = 400)	Odds Ratio	***p***-value
BMI	24.99 ± 5.75	23.21 ± 4.28	–	< 0.0001
WHR	0.99 ± 0.08	0.96 ± 0.13	–	0.008
*Blood Pressure (mm Hg)*				
Systolic BP (SBP)	142.90 ± 20.12	125.04 ± 8.30	–	< 0.0001*
Diastolic BP (DBP)	89.17 ± 11.13	83.46 ± 8.48	–	< 0.0001*
Pulse Pressure (PP)	53.73 ± 16.77	41.58 ± 8.12	–	< 0.0001*
Pulse Rate (PR)	82.11 ± 12.23	74.41 ± 4.91	–	< 0.0001*
Total Cholesterol (TC) (mg/dl)	172.75 ± 55.30	143.30 ± 27.87	–	< 0.0001*
Triglycerides (TG) (mg/dl)	191.27 ± 68.39	166.57 ± 30.22	–	< 0.0001*
HDL- C (mg/dl)	40.67 ± 7.71	44.88 ± 6.93	–	< 0.0001*
LDL-C (mg/dl)	121.29 ± 61.56	112.42 ± 33.94	–	0.01*
HDL/TC ratio	0.259 ± 0.10	0.327 ± 0.91	–	< 0.0001*
*Smoking*				
Smokers	159 (39.75%)	50 (12.5%)	4.62 (3.23–6.60)	< 0.0001*
Non-smokers	241 (60.25%)	350 (87.5%)	Ref. (1)	–
*Physical inactivity*				
Yes	249 (62.25%)	163 (40.75%)	2.40 (1.80–3.18)	< 0.0001*
No	151 (37.75%)	237 (59.25%)	Ref. (1)	–
*Dwelling*				
Urban	215 (53.75%)	158 (39.5%)		0.00005*
Rural	185 (46.25%)	242 (60.5%)		

* Significant values

*Abbrevations*: *BMI* Body Mass Index, *WHR* Waist Hip Ratio, *HDL-C* High Density Lipoprotein-Cholesterol, *LDL-C* Low Density Lipoprotein-Cholesterol

### Genetic polymorphisms

**Fig. 1:**
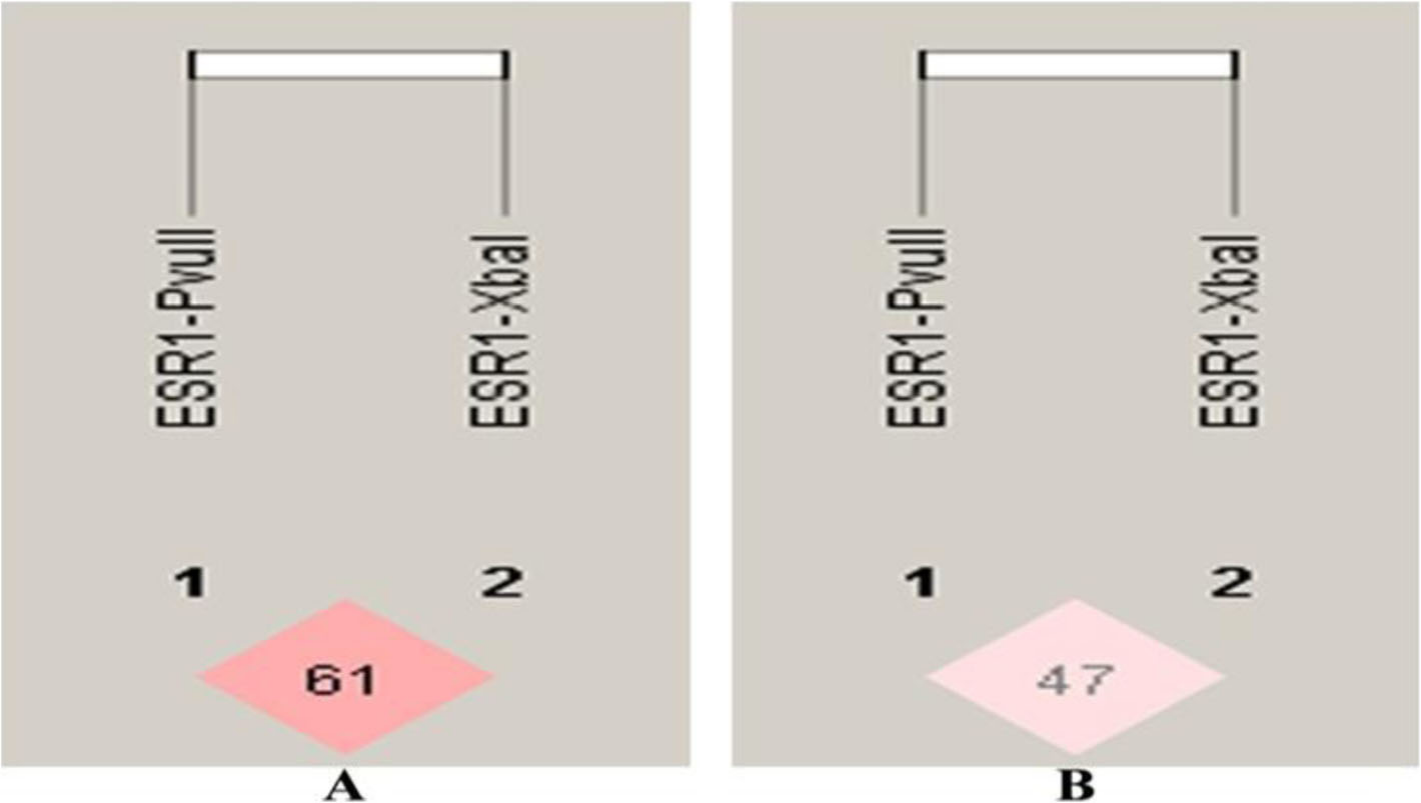
Linkage Disequilibrium (LD) plot for *ESR1* gene polymorphisms (**a**) Patients (**b**) Controls. [The numbers inside every box represent D’ values (%) of the LD]

**Fig. 2:**
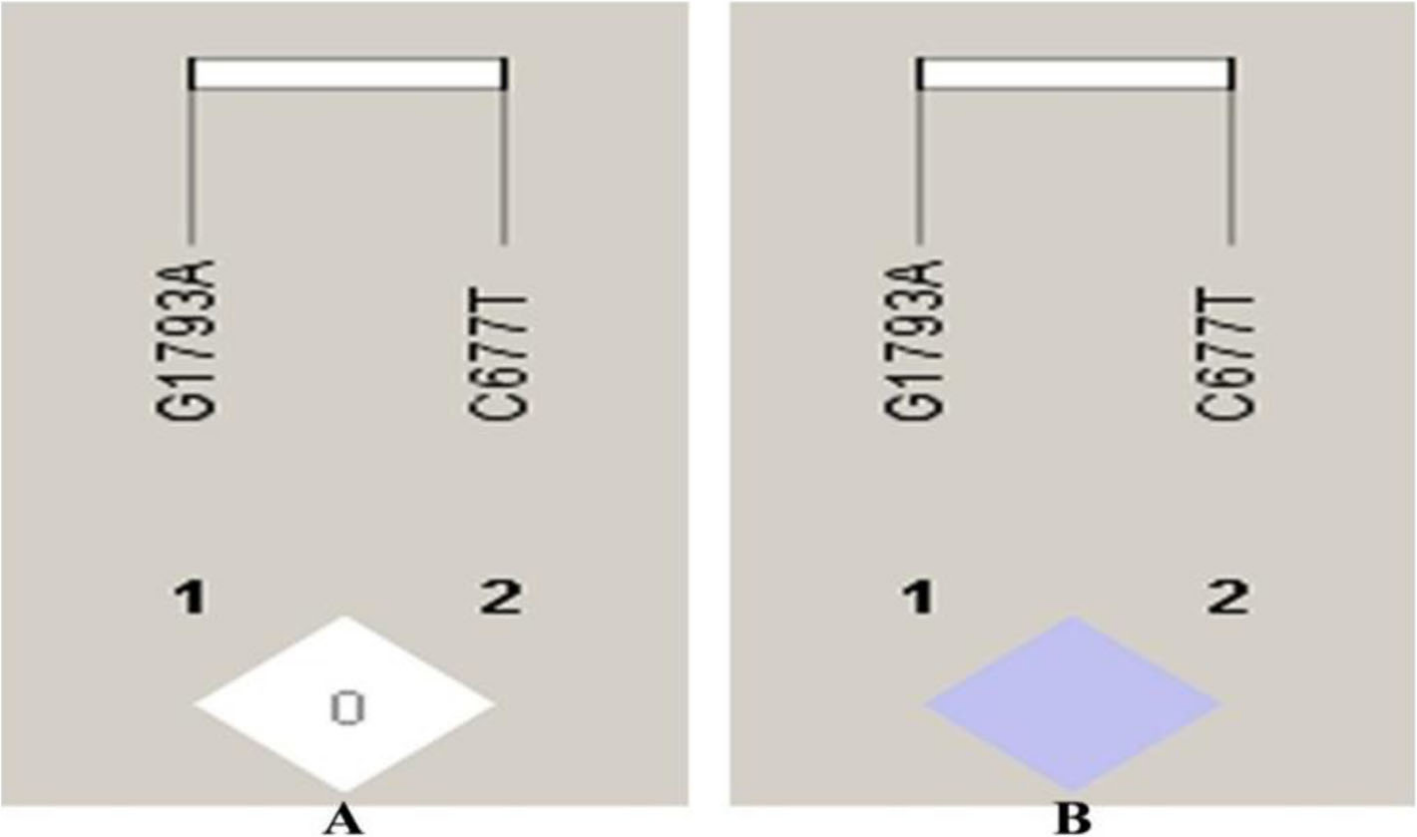
Linkage Disequilibrium (LD) plot for *MTHFR* gene polymorphisms (**a**) Patients (**b**) Controls. [The numbers inside every box represent D’ values (%) of the LD]

The distribution of allele frequencies and genotype percentage values for selected single nucleotide polymorphisms have been tabulated in Table 3. All the distributions were found to be in agreement with Hardy-Weinberg equilibrium (HWE) except for the patient group in *MTHFR* (rs1801133) polymorphism which could be attributed to very low frequency of diseases allele in Indian populations. Co-dominant, dominant, recessive, and allelic genetic models were applied to test the associations of the above said polymorphisms with CAD risk (Table 4). For *ESR1*(rs9340799) SNP, the variant G-allele was adding about 1.4 folds of risk; for *MTHFR* (rs1801133) SNP, T-allele was adding 5.3 folds risk and for *MS* (rs185087) SNP, the G-allele was conferring nearly 1.5 folds risk towards the susceptibility of CAD. No significant association was found under any of the tested genetic model for *ESR1*- (rs2234693), *CETP-*(rs708272) and *MTHFR* (rs2274976) SNPs. Distribution of haplotype frequencies of *ESR1* and *MTHFR* gene polymorphisms among CAD cases and healthy controls is depicted in Table 5. For *ESR1* gene SNPs, the haplotype C-G was found to confer approximately 5-fold risk [OR = 4.906 (3.604–6.678), *p* = 5.00e-015] and T-A about 1.6 fold risk [OR = 1.616 (1.318–1.980), *p* = 3.61e-006] of CAD outcome in the studied population. Regarding SNPs in *MTHFR* gene, haplotype T-G provided 4.7 fold risk [OR = 4.71 (2.270–9.762), *p* = 5.18e-006] of CAD development while haplotype C-G was found attributing 1.7 fold protection (1/0.57) towards the disease [OR = 0.57 (0.42–0.77), *p* = 0.0002]. The linkage disequilibrium (LD) pattern for *ESR1* and *MTHFR* gene polymorphisms has been depicted in Figs. 1 and 2. Based on measure of LD, the two SNPs for *ESR1* gene were in slight LD (as per D’ = 0.612, LOD = 17.88, r^2^ = 0.204) in patients but not in controls (D’ = 0.471, LOD = 5.07, r^2^ = 0.078) whereas the two *MTHFR* variants were not in LD (D’ = 0.007, LOD = 0, r^2^ = 0.000) in patients but complete LD was observed in controls (D’ = 1, LOD = 0.36, r^2^ = 0.001).

**Table 3:** Showing genotypic and allelic distribution of selected gene polymorphisms among cases and controls

Genotypic/Allelic frequencies	Cases (***N*** = 400)	Controls (***N*** = 400)
***ESR1*IVS1–397 T/C**		
TT	168 (42%)	175 (43.75%)
TC	187 (46.75%)	185 (46.25%)
CC	45 (11.25%)	40 (10%)
T (Major)	0.65	0.67
C (Minor)	0.35	0.33
**χ**^**2**^	0.65	0.5
p-value	0.77	0.4
***ESR1*- IVS1–351 A/G**		
AA	105 (26.25%)	133 (33.25%)
AG	196 (49%)	202 (50.5%)
GG	99 (24.75%)	65 (16.25%)
A (Major)	0.51	0.59
G (Minor)	0.49	0.42
**χ**^**2**^	0.16	0.7
p-value	0.64	0.4
***CETP*-(C277T) (TaqIB)**		
B2B2	104 (26%)	116 (29%)
B1B2	215 (53.75%)	212 (53%)
B1B1	81 (20.25%)	72 (18%)
B2 (Major)	0.53	0.56
B1 (Minor)	0.47	0.45
**χ**^**2**^	2.47	0.1
p-value	2.13	0.1
***MTHFR*C677T**		
CC	358 (89.5%)	391 (97.75%)
CT	38 (9.5%)	9 (2.25%)
TT	4 (1%)	0
C (Major)	0.94	0.98
T (Minor)	0.06	0.02
**χ**^**2**^	6.1	0.01*
p-value	0.05	0.82
***MTHFR*G1793A**		
GG	311 (77.75%)	330 (82.5%)
GA	85 (21.25%)	70 (17.5%)
AA	4 (1%)	0
G (Major)	0.88	0.9
A (Minor)	0.12	0.1
**χ**^**2**^	0.47	0.06
p-value	3.68	0.5
***MS*A2756G**		
AA	251 (62.75%)	294 (73.5%)
AG	139 (34.75%)	103 (25.75%)
GG	10 (2.5%)	3 (0.75%)
A (Major)	0.8	0.86
G (Minor)	0.2	0.14
**χ**^**2**^	3.32	0.1
p-value	3.54	0.06

* Significant values

**Table 4:** Depicting Odd Ratio (OR) and corresponding *p*-value of selected gene polymorphisms with CAD

MODEL	OR (95% CI)	*p*-value
ESR1 IVS1–397 T/C		
Co-dominant		
TC vs TT	1.05 [0.79–1.41]	0.7
CC vs TT	1.17 [0.73–1.88]	0.5
Dominant		
TC + CC vs TT	1.07 [0.81–1.42]	0.6
Recessive		
CC vs TC + TT	3.39 [0.92–12.42]	0.6
Allelic		
C vs T	1.07 [0.87–1.31]	0.5
ESR1- IVS1–351 A/G		
Co-dominant		
AG vs AA	1.23 [0.89–1.70]	0.2
GG vs AA	1.93 [1.29–2.89]	0.001*
Dominant		
AG + GG vs AA	1.40 [1.03–1.90]	0.03*
Recessive		
GG vs AG+ AA	1.70 [1.20–2.40]	0.003*
Allelic		
G vs A	1.37 [1.12–1.67]	0.002*
CETP-(C277T) (TaqIB)		
Co-dominant		
B1B2 vs B2B2	1.13 [0.82–1.57]	0.4
B1B1 vs B2B2	1.25 [0.83–1.90]	0.3
Dominant		
B1B2 + B1B1 vs B2B2	1.63 [0.85–1.59]	0.3
Recessive		
B1B1 vs B1B2 + B2B2	1.16 [0.81–1.64]	0.4
Allelic		
B1 vs B2	1.11 [0.91–1.35]	0.3
MTHFR C677T		
Co-dominant		
CT vs CC	4.61 [2.20–9.67]	0.00001*
TT vs CC	Not possible†	–
Dominant		
CT + TT vs CC	Not possible†	–
Recessive		
TT vs CT + CC	Not possible†	–
Allelic		
T vs C	5.36 [2.45–10.62]	< 0.0001*
MTHFR G1793A		
Co-dominant		
GA vs GG	1.29 [0.91–1.83]	0.15
AA vs GG	Not possible†	–
Dominant		
GA + AA vs GG	Not possible†	–
Recessive		
AA vs GA+ GG	Not possible†	–
Allelic		
A vs G	1.37 [1.00–1.90]	0.06
MS A2756G		
Co-dominant		
AG vs AA	1.58 [1.16–2.14]	0.003*
GG vs AA	3.90 [1.06–14.34]	0.03*
Dominant		
AG + GG vs AA	1.64 [1.22–2.22]	0.001*
Recessive		
GG vs AG+ AA	3.39 [0.93–12.42]	0.1
Allelic		
G vs A	1.57 [1.20–2.05]	0.001*

* Significant values

**Table 5:** Association of *ESR1* and *MTHFR* gene haplotypes with risk of CAD

Variant *ESR1* IVS1–397 T > C/ -351 A > G	Haplotype frequencies		OR (95% CI)	*p*-value^†^
	Patients (*n* = 400)	Controls (*n* = 400)		
C-A	0.068	0.258	0.210 [0.153–0.289]	2.22e-016*
C-G	0.278	0.073	4.906 [3.604–6.678]	5.00e-015*
T-A	0.439	0.327	1.616 [1.318–1.980]	3.61e-006*
T-G	0.215	0.342	0.525 [0.420–0.656]	1.27e-008*
Variant *MTHFR* C677T/ G1793A				
C-A	0.109	0.087	1.29 [0.93–1.79]	0.1
C-G	0.833	0.901	0.57 [0.42–0.77]	0.0002
T-G	0.050	0.011	4.71 [2.27–9.76]	5.18e-006*
T-A	0.007	0.000	–	–

* Significant values; ^†^Fisher’s *p*-value

### Gene –gene and gene –environment interaction

**Fig. 3:**
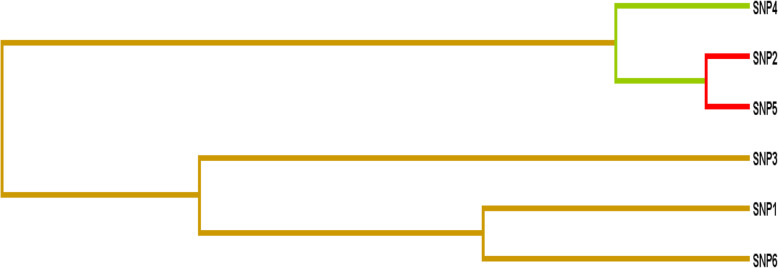
Interaction dendrogram depicting interaction between selected genetic variants among CAD cases and controls from population of Jammu region, Jammu and Kashmir State

Table 6 summarizes the results of the MDR analysis evaluated for gene-gene and gene environment interaction among cases and controls studied for the risk of developing CAD. A significant association of 2-loci (SNP 2, 5), 3-loci (SNP2, 3, 5), 5-loci (SNP 1, 2, 3, 5, 6) and 6-loci (SNP 1, 2, 3, 4, 5, 6) interactions was observed among total CAD cases. Entropy dendrogram (Fig. 3) revealed strong synergistic interaction between SNP 2 and SNP 5 (*ESR1* rs9340799 and *MTHFR* rs2274976) thus conferring risk of CAD. The MDR analysis did not reveal any highly redundant interaction between the studied polymorphisms; however, a moderate redundant effect was observed between SNP 4 SNP 2 and SNP 5 (*MTHFR* rs1801133, *ESR1* rs9340799 and *MTHFR* rs2274976, respectively) in the present population.

**Table 6:** Interaction analysis (MDR) among CVD cases and controls

Interaction Model	TBA	CVC	***p***-value
*Gene-Gene Interaction*			
**SNP 6**	0.52	8/10	0.001
**SNP 2, 5**	0.5948	10/10	< 0.0001*
**SNP 2, 3, 5**	0.6574	9/10	< 0.0001*
**SNP 1, 2, 3, 5**	0.6563	6/10	< 0.0001*
**SNP 1, 2, 3, 5, 6**	0.7111	9/10	< 0.0001*
**SNP 1, 2, 3, 4, 5, 6**	0.7186	10/10	< 0.0001*
*Gene-Environment Interaction*			
**A1**	0.6363	10/10	< 0.0001*
**SNP 5, A1**	0.6347	6/10	< 0.0001*
**SNP 2, 5, A1**	0.705	10/10	< 0.0001*
**SNP 1, 2, 5, A1**	0.7153	10/10	< 0.0001*
**SNP 1, 2, 3, 5, A1**	0.7103	7/10	< 0.0001*
**SNP 1, 2, 3, 5, 6, A1**	0.7301	9/10	< 0.0001*
**SNP 1, 2, 3, 5, 6, A1, A2**	0.7379	7/10	< 0.0001*
**SNP 1, 2, 3, 4, 5, 6, A1, A2**	0.7403	10/10	< 0.0001*

* Significant values. A1: Smoking; A2: Physical Inactivity

**Fig. 4:**
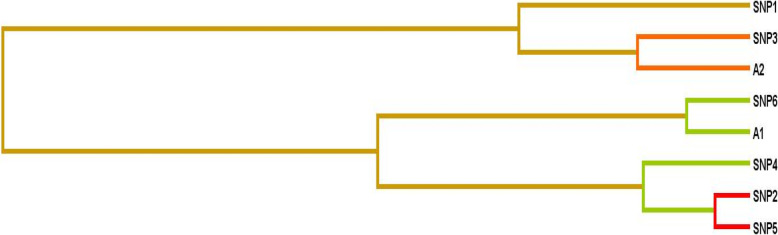
Interaction dendrogram depicting interaction between selected genetic variants and non-genetic factors among CAD cases and controls from population of Jammu region, Jammu and Kashmir State. (A1: smoking, A2: physical inactivity)

For the gene–environment dataset, the three-factor interaction model of SNP 2, 5 and smoking and four-factor model i.e. SNP 1, 2, 5 and Smoking were the best models identified with a maximum CVC of 10/10 and a highest testing balance accuracy of 70.5 and 71.53% respectively which was statistically significant as determined by 1000-fold permutation testing. The best interaction dendrogram for the assessment of gene-environment interactions was generated through MDR (Fig. 4). Moderately synergistic interaction was observed between SNP 3 (*CETP* rs708272) and physical inactivity whereas weak correlation was present between SNP 6 (*MS* rs185087) and smoking.

## Discussion

The findings of the present study provide a comprehensive understanding of association of *ESR1*, *CETP*, *MTHFR* and *MS* gene polymorphisms with the outcome of CAD in population of Jammu region (JandK). ESR1 gene encodes for estrogen receptor 1 or ER-α which acts as a ligand activated transcription factor involved in imparting estrogen actions. The two *ESR1* gene polymorphisms considered in the present study are *PvuII* (rs2234693) and *XbaI* (rs9340799)*.* The *PvuII* polymorphism involves 454–397 T > C transition in such an element of intron 1 which may affect the binding of the transcription factor, resulting in the alteration of protein expression of target gene [16] whereas *XbaI* polymorphism is the result of A to G transition in intron 1 of the gene, approximately 351 base pairs upstream of exon 2. The clear functional picture of *ESR1*-*XbaI* polymorphism is still under exploration. The allele frequencies of *ESRI-PvuII* variants in the present study are in accordance with previous findings in population of Jammu [17] whereas these frequencies have been found to be higher when compared to adjoining North-west Indian populations [18]. The present study did not find any association of *ESR1*-(rs2234693) polymorphism with CAD in the studied population of Jammu region. Our study is in concordance with other studies done by Kjaergaard et al.*,* [7], Koch et al.*,* [19], Boroumand et al.*,* [20], Wu et al.*,* [21], Boroumand et al.*,* [22], Rebelo et al.*,* [23], Jiang et al.*,* [24] and Mariah et al.*,* [25]. In the study under reference, the authors considered a positive effect of *ESR1*- (rs9340799) polymorphism with CAD occurrence in population of Jammu region (JandK). The impact of *ESR1*-(rs9340799) polymorphism has remained conflicting with respect to disease susceptibility in populations worldwide and in contrast to our results, contradictory observations have been given by Koch et al.*,* [19], Mariah et al.*,*[25], Huang et al.*,* [26], Yilmaz et al.*,* [27], Munshi et al.*,* [28] and Wei et al.*,* [29].

Studying genetic association by approach of haplotypes generate more powerful analysis in case-control association studies. The two SNPs of *ESR1* gene were observed to be in slight LD in patients but not in controls. Majority of haplotype combinations which were suggesting a defending role against CAD contain rs2234693 C-allele and rs9340799 A-allele of *ESR1* gene. It is reported that the C-allele of *ESR1*-*PvuII* and its associated genotypes and haplotypes are inversely and consistently associated with obesity [30]. Similar to our findings, T-A haplotype was found to be associated with an increased risk of severe preeclampsia among Caucasian women [31] and hyperlipidemia in Chinese Han women [16] whereas, on the contrary, lack of association with any of the haplotype combination towards CAD was delineated by Koch et al.*,* [19] and Rebelo et al.*,* [23].

The genetic variation of *CETP* is a major determinant of inter-individual variation in susceptibility to CAD. The *CETP*-*TaqIB* (rs708272) polymorphism may contribute to recurrent risk of CAD, possibly via abnormal HDL-C remodeling and by spoiling anti-atherogenic properties of HDL-C. The results of the present study point towards a non- significant association of *CETP*-(rs708272) polymorphism with risk of CAD in the population of Jammu region (JandK). The allele and genotypic frequencies observed in the present study are comparable with previous findings [32–35]. Our results are consistent with findings of other studies which have revealed that *CETP*-(rs708272) polymorphism was not associated with CAD [35–37]. In contrast to our results, positive association was reported by Bhanushali and Das [38] Rahimi et al.*,* [39], Kaman et al.*,* [40] and Iwanicka et al.*,* [41].

Increased circulating levels of homocysteine (sulphur-containing amino acid) accerlate atherosclerosis by several mechanisms. It is involved in the promotion of platelet activation, hypercoagulability, formation of hydrogen peroxide and oxidative stress, endothelial dysfunction, smooth muscle cell proliferation, oxidation and peroxidation of lipids [42]. Two genes namely Methylenetetrahydrofolate reductase (*MTHFR*) and Methionine synthase (*MS/MTR*) are considered to be critical in homocysteine metabolic pathway. *MTHFR* gene catalyses the conversion of 5,10- methylenetetrahydrofolate (5,10-MTHF) into 5-methyltetrahydrofolate (5-MTHF). The methyl group of 5-MTHF is given to Hcy to form methionine by *MS* gene via remethylation reaction. The allelic and genotypic frequencies for *MTHFR* (rs1801133) polymorphism as reported in the study under reference are consistent with previous studies [43–45]. The mutant genotype was found absent in controls in our study. Likewise, Raina et al.*,* [9], Markan et al.*,* [43], Lakshmi et al.*,* [44], Matam et al.*,* [45], Raina et al.*,* [46] did not record any mutant genotype in controls in their study. We reported a significant association of the said polymorphism and the results are consistent with earlier investigations [9, 43, 44, 47, 48]. On the contrary, it was identified in a study that homozygosity of the T allele was a significant protective factor against CAD [49]. Rady et al.*,* [50] reported a novel polymorphic site (G1793A) in exon 11 of *MTHFR* gene that results in an arginine to glutamine substitution at codon 594 (R594Q). The functional aspect of G1793A polymorphism (rs2274976) on MTHFR activity, homocysteine levels and risk of CVD is under exploration. The results of our study demonstrate that the *MTHFR* (rs2274976) is not in association with CAD in population of Jammu region which is in accordance with other studies done by Trifonova et al.*,* [51] and Neto et al.*,* [52]. The combinatorial effect of *MTHFR* (rs1801133 and rs2274976) polymorphisms towards CAD has not been studied so far. The LD scores assessed by pair-wise comparison of two *MTHFR* polymorphisms suggest that the two SNPs are not in LD in cases whereas controls are showing complete LD. The haplotype T-G is observed to confer 4.7-fold risk towards CAD whereas haplotype C-G provides nearly 1.7 fold protection towards development of CAD in studied population of Jammu region. The present study affirms that G-allele and GG- genotype of *MS* gene polymorphism (rs185087) is add significantly to the risk towards susceptibility of CAD as has been documented earlier [9, 53, 54].

Several large cross-sectional surveys have been conducted in India in the past twenty years that provide a brief summary of identified risk factors associated with CAD. During the present study, various risk factors were reviewed and recorded to identify their potential and contribution in the development of CAD. The risk factors such as cigarette smoking, sedentary lifestyle and elevated blood pressure contribute majorly toward the development of CVD in an individual. The present study also depicts a significant difference between lipid levels and BMI in patients as compared to controls. These findings are in line with previous studies done by Iyer et al.*,* [55], Raina et al.*,* [56], Kalra et al.*,* [57], Sekhri et al.*,* [58], Koju et al.*,* [59].

The gene-gene interaction dendrogram indicates that *ESR1* (rs9340799) and *MTHFR* (rs2274976) are conferring strong predisposition and association with the CAD which can be attributed to the role of both the genes in anti- atherosclerotic events. Furthermore, gene-environment interaction has revealed the combined effect of lack of physical activity and *CETP* (rs708272) polymorphism towards the development of CAD as both these factors effect altered lipid metabolism. The current study needs to be elaborated further for determining the role of these polymorphisms at transcriptome and proteome levels to elucidate the disease etiology and susceptibility.

## Conclusion

To conclude, the findings of the present study depict a significant involvement of *ESR1* (rs9340799), *MTHFR* (rs1801133) and *MS* (rs185087) gene polymorphisms in the CAD susceptibility among the inhabitants of Jammu region. The lack of association of other genetic polymorphisms with CAD in the present study may be attributed to expression of population specific influence as well as locus heterogeneity and allelic heterogeneity.

## Data Availability

We declare that the data and the material used in the manuscript under reference are with the corresponding author and can be reproduced / shared as and when asked for by the editorial team of BMC Cardiovascular Disorders in connection with the publishing of the manuscript entitled “Association of *ESR1* (rs2234693 and rs9340799), *CETP* (rs708272), *MTHFR* (rs1801133 and rs2274976) and *MS* (rs185087) polymorphisms with Coronary Artery Disease (CAD)”.

## Abbreviations

BMI: Body Mass Index
CAD: Coronary artery disease
CVDs: Cardiovascular diseases
CVC: Cross-validation consistency
DBP: Diastolic Blood Pressure
D’: Lewontin’s coefficient
HDL-C: High Density Lipoprotein-Cholesterol
HWE: Hardy-Weinberg equilibrium
J and K: Jammu and Kashmir
LD: Linkage Disequilibrium
LDL-C: Low Density Lipoprotein-Cholesterol
LOD: log of the likelihood of odds ratio
MDR: Multifactor Dimensionality Reduction
MI: Myocardial Infarction
PCR-RFLP: Polymerase Chain Reaction- Restriction Fragment Length Polymorphism
PP: Pulse Pressure
PR: Pulse Rate
SBP: Systolic Blood Pressure
TBA: Testing balance accuracy
TC: Total Cholesterol
TG: Triglycerides
UT: Union Territory
WHR: Waist Hip Ratio

## References

[1] Heart disease and stroke statistics, 2019 update. (www.ahajournals.org/doi/10.1161)..

[2] Ardeshna DR, Bob-Manuel T, Nanda A, Sharma A (2018). Asian-Indians: a review of coronary artery disease in this understudied cohort in the United States. Ann Transl Med.

[3] Liu H, Pedram A, Kim JK (2011). Oestrogen prevents cardiomyocyte apoptosis by suppressing p38α-mediated activation of p53 and by down-regulating p53 inhibition on p38β. Cardiovasc Res.

[4] Gao HH, Gao LB, Wen JM (2014). Genetic polymorphisms in the ESR1 gene and cerebral infarction risk: a meta-analysis. DNA Cell Biol.

[5] Murphy E (2011). Estrogen signaling and cardiovascular disease. Circ Res.

[6] Schuit SCE, Oei HHS, Witteman JCM, van Kessel CHG (2004). Estrogen receptor α gene polymorphisms and risk of myocardial infarction. JAMA..

[7] Kjaergaard AD, Ellervik C, Tybjærg-Hansen A, Axelsson CK, Grønholdt MLM, Grande P (2007). Estrogen receptor α polymorphism and risk of cardiovascular disease, cancer, and hip fracture cross-sectional, cohort and case-control studies and a meta-analysis. Circulation..

[8] Hassanzadeh T, Firoozrai M, Zonouz AE, Zavarehee A, Paoli M (2009). *Taq1*B polymorphism of cholesteryl ester transfer protein (CETP) gene in primary combined hyperlipidaemia. Indian J Med Res.

[9] Raina JK, Sharma M, Panjaliya RK, Bhagat M, Sharma R, Bakaya A, Kumar P (2016). Methylenetetrahydrofolate reductase C677T and methionine synthase A2756G gene polymorphisms and associated risk of cardiovascular diseases: a study from Jammu region. Indian Heart J.

[10] WHO Expert Consultation (2004). Appropriate body-mass index for Asian populations and its implications for policy and intervention strategies. Lancet..

[11] Executive Summary of the Third Report of the National Cholesterol Education Program (NCEP) (2001). Expert Panel on Detection, Evaluation, and Treatment of High Blood Cholesterol in Adults (Adult Treatment Panel III). JAMA.

[12] Chobanian AV, Bakris GL, Black HR, Cushman WC, Green LA, Izzo JL, Jones DW (2003). The seventh report of the joint National Committee on prevention, detection, evaluation, and treatment of high blood pressure: the JNC 7 report. JAMA..

[13] Sambrook J, Russell DW (2001). Molecular cloning, a laboratory manual.

[14] Skol AD, Scott LJ, Abecasis GR, Boehnke M (2006). Joint analysis is more efficient than replication-based analysis for two-stage genome-wide association studies. Nat Genet.

[15] Ritchie MD, Hahn LW, Roodi N, Bailey LR, Dupont WD, Parl FF, Moore JH (2001). Multifactor dimensionality reduction reveals high-order interactions among estrogen metabolism genes in sporadic breast cancer. Am J Hum Genet.

[16] Zhao T, Zhang D, Liu Y, Zhou D, Chen Z, Yang Y (2010). Association between ESR1 and ESR2 gene polymorphisms and hyperlipidemia in Chinese Han postmenopausal women. J Hum Genet.

[17] Panjaliya RK, Gupta D, Raina JK, Bhardwaj R, Gupta A, Kumar P (2013). Study of genetic variation of *Alu* CD4, LPL-*PvuII* and ESR1-*PvuII* in different population groups of Jammu region (JandK). Int J Biol Biomed Sci.

[18] Saini JS, Kumar A, Matharoo K, Sokhi J, Badaruddoza BAJS (2012). Genomic diversity and affinities in population groups of north West India: an analysis of *Alu* insertion and a single nucleotide polymorphism. Gene..

[19] Koch W, Hoppmann P, Pfeufer A, Mueller JC, Schömig A, Kastrati A (2005). No replication of association between estrogen receptor α gene polymorphisms and susceptibility to myocardial infarction in a large sample of patients of European descent. Circulation..

[20] Boroumand M, Ghaedi M, Mohammadtaghvaei N, Pourgholi L, Anvari MS, Davoodi G (2009). Association of estrogen receptor α gene polymorphism with the presence of coronary artery disease documented by coronary angiography. Clin Biochem.

[21] Wu MM, Hsieh YC, Lien LM, Chen WH, Bai CH, Chiu HC (2010). Association of estrogen receptor (alpha) genotypes/haplotypes with carotid intima-media thickness in Taiwanese women. Angiology..

[22] Boroumand M, Ghasemi Y, Shirani S, Pourgholi L, Anvari MS, Sepehriseresht S (2011). Association between estrogen receptor-alpha *PvuII* and *XbaI* gene polymorphisms with extracranial carotid stenosis. Labmedicine..

[23] Rebelo AC, Verlengia R, Kunz V, Tamburus N, Cerda A, Hirata R (2012). Lack of association of estrogen receptor alpha gene polymorphisms with cardiorespiratory and metabolic variables in young women. Int J Mol Sci.

[24] Jiang N, Yang G, Peng CL (2015). ESR1 gene polymorphisms *Pvu*II (rs2234693T>C) and *Xba*I (rs9340799A>G) may not be directly correlated with cardiovascular disease risk. Genet Mol Res.

[25] Mariah RA, Baghdadi H, El-din Ahmed K, Mostafa N, Ayat MMA, Nansour T (2016). Frequency of estrogen receptor-1 (ESR-1) gene polymorphism (PvuII and XbaI) in patients with coronary artery disease. Am J Med Biol Res.

[26] Huang Q, Wang TH, Lu WS, Mu PW, Yang YF, Liang WW (2006). Estrogen receptor alpha gene polymorphism associated with type 2 diabetes mellitus and the serum lipid concentration in Chinese women in Guangzhou. Chin Med J.

[27] Yilmaz A, Menevse S, Erkan AF, Ergun MA, Ilhan MN, Cengel A (2007). The relationship of the ESR1 gene polymorphisms with the presence of coronary artery disease determined by coronary angiography. Genet Test.

[28] Munshi A, Sharma V, Kaul S, Al-Hazzani A, Alshatwi AA, Manohar VR (2010). Estrogen receptor α genetic variants and the risk of stroke in a south Indian population from Andhra Pradesh. Clin Chim Acta.

[29] Wei CD, Zheng HY, Wu W, Dai W, Tong YQ, Wang M (2013). Meta-analysis of the association of the rs2234693 and rs9340799 polymorphisms of estrogen receptor alpha gene with coronary heart disease risk in Chinese Han population. Int J Med Sci.

[30] Goulart AC, Zee RYL, Rexrode KM (2009). Estrogen receptor 1 gene polymorphisms and decreased risk of obesity in women. Metabolism..

[31] Molvarec A, Ver A, Fekete A, Rosta K, Derzbach L, Derzsy Z (2007). Association between estrogen receptor α (ESR1) gene polymorphisms and severe preeclampsia. Hypertens Res.

[32] Dixit M, Mittal B (2005). Frequencies of CETP gene *Taq*I B and D442G polymorphisms in north Indian population. Curr Sci.

[33] Padmaja N, Kumar MR, Soya SS, Adithan C (2007). Common variants of cholesteryl ester transfer protein gene and their association with lipid parameters in healthy volunteers of Tamilian population. Clin Chim Acta.

[34] Tantray JA, Kumar YS, Jamil K (2013). Pharmacogenomic studies of cholesteryl ester transfer protein (CETP) genotypes in suspected CAD patients. Int J Pharm Sci Res.

[35] Gundogdu F, Gurlertop Y, Pirim I, Sevimli S, Dogan H, Arslan S (2009). The relationship between genetic variations of the cholesteryl ester transfer protein gene and coronary artery disease in Turkish subjects. Eurasian J Med.

[36] Kaestner S, Patsouras N, Spathas DH, Flordellis CS, Manolis AS (2010). Lack of association between the cholesteryl ester transfer protein gene-*TaqIB* polymorphism and coronary restenosis following percutaneous transluminal coronary angioplasty and stenting: a pilot study. Angiology..

[37] Lu Y, Tayebi N, Li H, Saha N, Yang H, Heng CK (2013). Association of CETP Taq1B and -629C > a polymorphisms with coronary artery disease and lipid levels in the multi-ethnic Singaporean population. Lipids Health Dis.

[38] Bhanushali AA, Das BR (2010). Genetic variants at the APOE, lipoprotein lipase (LpL), cholesteryl ester transfer protein (CETP), and endothelial nitric oxide (eNOS) genes and coronary artery disease (CAD): CETP Taq1 B2B2 associates with lower risk of CAD in Asian Indians. J Community Genet.

[39] Rahimi Z, Nourozi-Rad R, Rahimi Z, Parsian A (2012). Strong interaction between T allele of endothelial nitric oxide synthase with B1 allele of cholesteryl ester transfer protein TaqIB highly elevates the risk of coronary artery disease and type 2 diabetes mellitus. Human Genomics.

[40] Kaman D, İlhan N, İlhan N, Akbulut M (2015). *TaqIB* and severity of coronary artery disease in the Turkish population: a pilot study. Bosn J Basic Med Sci.

[41] Iwanicka J, Iwanicki T, Niemiec P, Balcerzyk A, Krauze J, Gorczyńska-Kosiorz S (2018). Relationship between *CETP* gene polymorphisms with coronary artery disease in polish population. Mol Biol Rep.

[42] Ilhan N, Kucuksu M, Kaman D, Ilhan N, Ozbay Y (2008). The 677 C/T MTHFR polymorphism is associated with essential hypertension, coronary artery disease, and higher homocysteine levels. Arch Med Res.

[43] Markan S, Sachdeva M, Sehrawat BS, Kumari S, Jain S, Khullar M (2007). MTHFR 677 CT/MTHFR 1298 CC genotypes are associated with increased risk of hypertension in Indians. Mol Cell Biochem.

[44] Lakshmi SVV, Naushad SM, Rupasree Y, Rao DS, Kutala VK (2011). Interactions of 5′ UTR Thymidylate synthase polymorphism with 677 C/T methylene Tetrahydrofolate Reductase and 66 a/G Methyltetrahydrofolate Homocysteine methyl- Transferase Reductase polymorphisms determine susceptibility to coronary artery disease. J Atheroscler Thromb.

[45] Matam K, Khan IA, Hasan Q, Rao P. Coronary artery disease and the frequencies of MTHFR and PON1 gene polymorphism studies in a varied population of Hyderabad, Telangana region in south India. J King Saud Univ Sci. 2014. 10.1016/j.jksus.2014.09.002.

[46] Raina JK, Panjaliya RK, Sharma M, Bhardwaj R, Bakaya A, Kumar P (2016). Methylenetetrahydrofolate reductase C677T gene polymorphism and predisposition to essential hypertension. Int J Genetics.

[47] Dhar S, Chatterjee S, Ray S, Dutta A, Sengupta B, Chakrabarti S (2010). Polymorphisms of methylenetetrahydrofolate reductase gene as the genetic predispositions of coronary artery diseases in eastern India. J Cardiovasc Dis Res.

[48] Latheef K, Rajasekhar D, Vanajakshamma V, Aparna BR, Chaudhury A, Sarma PVGK (2018). Association of *MTHFR, IL-6* and *ICAM-1* gene polymorphisms with coronary artery disease in south-Indian ethnic subset: a case-control study. J Cardiovasc Disease Res.

[49] Butler S, Young A, Akam EC, Sinha N, Agrawal S, Mastana S (2018). Association of methylenetetrahydrofolate reductase (MTHFR) C677T and A1298C polymorphisms with coronary artery disease (CAD) in a north Indian population. Cogent Med.

[50] Rady PL, Szucs S, Grady J, Hudnall SD, Kellner LH, Nitowsky H (2002). Genetic polymorphisms of methylenetetrahydrofolate reductase (MTHFR) and methionine synthase reductase (MTRR) in ethnic populations in Texas; a report of a novel MTHFR polymorphic site, 1793G>a. Am J Med Genet.

[51] Trifonova EA, Spiridonova MG, Gabidulina TV, Urnov FD, Puzyrev VP, Stepanov VA (2012). Analysis of the MTHFR gene linkage disequilibrium structure and association of polymorphic gene variants with coronary atherosclerosis. Russ J Genet.

[52] Neto AIM, de Moura Júnior JR, Persuhn DC (2013). Frequency of MTHFR G1793A polymorphism in individuals with early coronary artery disease: cross-sectional study. Sao Paulo Med J.

[53] Eghlim FF, Ashavaid TF, Nair KG (2006). Genetic determinants of hyperhomocysteinemia in atherosclerosis. Indian J Clin Biochem.

[54] Jemaa R, Achouri A, Kallel A, Ali SB, Mourali S, Feki M (2008). Association between the *2756A> G* variant in the gene encoding methionine synthase and myocardial infarction in Tunisian patients. Clin Chem Lab Med.

[55] Iyer UM, Bhoite RM, Shah T (2011). Risk factor analysis in coronary heart diseases and identifying at risk patients using a simple risk score test. Asian J Exp Biol Sci.

[56] Raina JK, Sharma M, Sethi S, Panjaliya RK, Bakaya A, Kumar P (2018). A pilot study on recognition and prevalence of risk factors for cardiovascular diseases in north Indian populace of Jammu and Kashmir. J Hum Ecol.

[57] Kalra S, Narain S, Karki P, Ansari JA, Ranabhat K, Basnet N (2011). Prevalence of risk factors for coronary artery disease in the community in eastern Nepal- a pilot study. JAPI..

[58] Sekhri T, Kanwar RS, Wilfred R, Chugh P, Chhillar M, Aggarwal R (2014). Prevalence of risk factors for coronary artery disease in an urban Indian population. BMJ Open.

[59] Koju R, Humagain S, Khanal K (2014). Association of cardiovascular risk factors and coronary artery lesion among coronary artery disease patients. Kathmandu Univ Med J.

